# Stem cells for spinal cord regeneration: Current status

**DOI:** 10.4103/2152-7806.74240

**Published:** 2010-12-25

**Authors:** Zain A. Sobani, Syed A. Quadri, S. Ather Enam

**Affiliations:** Department of Neurosurgery, Aga Khan University Hospital, Stadium Road, P.O. Box 3500, Karachi 74800, Pakistan

**Keywords:** Mesenchymal stem cells [A11.872.580], Spinal cord injuries [C10.228.854.770], Stem cells [A11.872]

## Abstract

**Background:**

Nearly 11,000 cases of spinal cord injury (SCI) are reported in the United States annually. Current management options give a median survival time of 38 years; however, no rehabilitative measures are available. Stem cells have been under constant research given their ability to differentiate into neural cell lines replacing non functional tissue. Efforts have been made to establish new synapses and provide a conducive environment, by grafting cells from autologous and fetal sources; including embryonic or adult stem cells, Schwann cells, genetically modified fibroblasts, bone stromal cells, and olfactory ensheathing cells and combinations/ variants thereof.

**Methods:**

In order to discuss the underlying mechanism of SCI along with the previously mentioned sources of stem cells in context to SCI, a simple review of literature was conducted. An extensive literature search was conducted using the PubMed data base and online search engines and articles published in the last 15 years were considered along with some historical articles where a background was required.

**Results:**

Stem cell transplantation for SCI is at the forefront with animal and *in vitro* studies providing a solid platform to enable well-designed human studies. Olfactory ensheathing cells seem to be the most promising; whilst bone marrow stromal cells appear as strong candidates for an adjunctive role.

**Conclusion:**

The key strategy in developing the therapeutic basis of stem cell transplantation for spinal cord regeneration is to weed out the pseudo-science and opportunism. All the trials should be based on stringent scientific criteria and effort to bypass that should be strongly discouraged at the international level.

## INTRODUCTION

According to the spinal cord injury information network, 300,000 people suffer from spinal cord injury (SCI) in the United States and nearly 11,000 new cases are reported annually. The estimated odds of a traumatic spinal injury in the United States is 40 per million, and the estimated cost of these patients is US$ 8 billion annually, with individual costs of up to 1.35 Million over the course of their life. Epidemiological data regarding the prevalence of SCI internationally is unavailable; however, it is estimated that more than 130,000 individuals suffer from SCI every year. The average age of an SCI patient is 30 years, indicating that it incapacitates individuals in the prime of their life. The high incidence in young age groups is attributable to the large number of cases of road traffic accidents (RTA) and sports injuries in this group. However, no cure is currently available for these individuals.

Current emergency management of associated trauma and other cardio-pulmonary sequelae of SCI increase the life expectancy of the patient giving a median survival time of 38 years post injury. Forty three percent of SCI patients survive for at least 40 years; however, no rehabilitative measures are currently available. The lack of mobility and increased dependence of SCI patients causes increasing psychological stress along with secondary complications such as urinary tract infection and pressure sores, requiring constant hospitalization further burdening the health care system. Before we move on to the review, an overall understanding of the basic pathophysiology underlying the disease is essential.

SCI may result in severe sensory and motor deficits at and below the level of injury due to extensive neuronal loss, acute axonal damage, demyelination and scar formation. The severity of SCI ranges from incomplete myelopathy/paraparesis to complete paraplegia. The actual damage is exacerbated by the immune response to spinal trauma. The post traumatic microenvironment has been shown to cause apoptosis and damage to surrounding functional neurons. Any potential method for functional restoration must incorporate neuroprotective strategies along with the re-establishment of ascending/descending tracts and local synapses. Due to the limited capacity of axonal regeneration and the presence of local inhibitory factors, recovery is minimal.

## MECHANISM OF INJURY

### Primary and Secondary Phases of Injury

It has been established that the pathophysiology of acute SCI occurs in two stages; the initial impact coupled with persistent compression comprising the primary injury. Laceration, contusion, compression and concussion are hallmarks of primary injury caused by the initial physical and/or mechanical trauma to the spinal cord. The agents responsible for this can be penetrating foreign objects, bone fragments, hematoma, shock wave or a combination of these elements. Complete transection of the spinal cord is rare; residual connections usually persist after injury indicating potential for recovery. The insults incurred in response to the primary injury comprise the secondary injury giving rise to ischemia, micro-vascular damage, glutamatergic excitotoxicity, oxidative stress, and inflammation.

Most of the cell death occurring due to acute SCI is due to secondary mechanisms. A wave of secondary cell injury spreads rostrally and caudally from the site of impact causing structural and functional disturbance of the spinal cord. Cell death begins centrally; effecting cell bodies first as swelling of the cord within the confines of the spinal canal creates far greater pressure than venous blood pressure. As a result blood flow ceases depriving the cord interior of oxygen and nutrients, leading to delayed and progressive degeneration of the residual axonal tracts.

Degradation of white matter results in the loss of motor, sensory and autonomic functions along with abnormal sprouting that can cause spasticity and neuropathic pain. Major contributors to secondary injury are ischemia, lipid peroxidation, edema, increases in free radicals and excitotoxic levels of transmitters (glutamatergic excitotoxicity). The loss of oligodendrocytes and scarce expression of myelin-associated genes are more lethal than the loss of neurons.

### Role of Cytokines and Inflammatory Mediators

A study carried out on rats to note the changes in the microenvironment of the injured spinal cord revealed that during the acute phase of injury, expression of mRNA coding proinflammatory cytokines like interleukins (IL)-1a, IL-1b, IL-6 and tumor necrosis factor-a (TNF-a), increased 6 to 12 h after the injury and peaked at four days. These proinflammatory cytokines discussed below are known to exhibit cytotoxicity and induce apoptosis in neurons and oligodendrocytes.

#### IL-6 cytokine super family

Leukemia inhibitory factor (LIF) and ciliary neurotrophic factor (CNTF) belonging to the IL-6 cytokine super family are also known to stimulate the differentiation of neural stem/progenitor cells into astroglia by activating the gp130/Janus kinase (JAK)/signal transducers and STAT pathway during the acute phase of the injury. These reactive astroglia are known to express chondroitin sulfate proteoglycans (CSPG) which inhibits axonal regeneration. The mechanism of scar formation, inhibitory effect of scar on axonal regeneration and the role of CSPG is discussed in more detail later in this review article.

#### TNF/nerve growth factor receptor superfamily

Fas, a type I membrane protein belonging to the TNF/nerve growth factor receptor superfamily, mediates the induction of apoptosis by interacting with Fas ligand (FasL). The activation of FasL, a membrane-type cytokine belonging to the TNF family expressed in T cells, results in activation of caspases 3 and 8 leading to cell death and apoptosis The role of Fas in SCI has been highlighted by the facts that the number of cells expressing Fas was noted to increase within 72 h of SCI and also the implication of FasL antibodies improved neurological outcome after SCI.

#### Tissue plasminogen activator (TPA)

TPA, a serine protease and a well-recognized thrombolytic agent, is considered to be involved in demyelinating diseases by degrading myelin basic protein (MBP) and contributing to excitotoxic neuronal death by activating microglia. TPA’s mechanism of action strongly suggests that it has a possible role in secondary SCI by promoting demyelination through plasmin, which directly degrades MBP and initiates the matrix metalloproteinase (MMP) cascade known to play an important role in the breakdown of myelin sheaths. This is strongly supported by a decrease in the neural damage in TPA-deficient mice after spinal cord injury. MMPs, a large family of proteolytic enzymes, have been recognized to have an involvement in the events following a traumatic CNS injury. MMP-2 has been shown to promote functional recovery after SCI by regulating the formation of a glial scar. Pharmacological blockade of MMPs during the first three days after injury has been shown to improve functional recovery, but when the blockade was extended to seven days the improvement was lost. This has been attributed to the decreased expression of MMP-2. Increased levels of MMP-2 have been noted after transplantation of human umbilical cord blood stem cells(UCBSCs) in rats leading to a decrease in the glial scar size; however, the underlying mechanism is not yet clear.

### Axonal Degeneration and Demyelination

The distal section of a transected axon (which is isolated from the neuronal cell body) degenerates and is in turn phagocytosed. Prior to phagocytosis, there is disruption of the axonal membrane locally along with swelling of axonal mitochondria, formation of “nodal blebs,” loss of microtubules, loss of neurofilament side arms, and disconnection of axons. The proximal axon section typically survives but fails to regrow and reinnervate its targets. In the peripheral nervous system (PNS) spontaneous regeneration from the proximal part is a natural phenomenon but axons in the central nervous system (CNS) are devoid of this property; however, limited sprouting has been noted in spared axons contributing to some functional recovery. This lack of axonal regeneration is mainly explained by the presence of axonal growth inhibitors in the CNS. Myelin-associated proteins and the scar formed at the site of injury are also known to hinder axonal growth. The spared tissue (remaining axons) connects the spinal cord above and below the lesion and might in turn provide some functionality below the level of the lesion; however, demyelination of these axons often compromises their function as well.

Although the spared axons are not directly affected by the injury, their ability to conduct electrical impulses becomes less efficient due to lack of myelin sheaths. Ion channels have to be reshuffled in order to compensate for the demyelination, further impairing conduction velocity of action potentials. Persistently demyelinated axons are susceptible to degeneration as evident in multiple sclerosis in which axonal and neuronal loss is thought to be brought about by demyelination. Along with increasing conduction velocities across axons, myelin also plays a key role in determining the molecular organization of axons. The demyelinated axons are also susceptible to injury by the micro environment at the site of lesion.

### Oxidative Injury to Neurons and Oligodendrocytes

Neurons and oligodendrocytes are highly susceptible to secondary cell death after SCI. Neurons have a high rate of oxidative metabolism and contain lower levels of antioxidants such as glutathione when compared to astroglial cells. Their high rate of oxidative metabolism makes them prone to injury by reactive oxygen species following ischemia. Phase II enzymes including g-glutamyl-cysteine synthase, quinone reductase, glutathionetransferase, epoxide hydrolase, UDP-glucoronosyltransferase play an important role, either directly or indirectly in neutralization of damaging free radicals and protect the cell from oxidative injury. However, neurons respond differently to molecular mechanisms involving the activation of Phase II enzymes. T-butylhydroquinone, a phase II enzyme inducer, has been shown to increase glutathione levels along with glutathione reductase and thioredoxin reductase activity levels in cortical astrocytes, but not in neurons.

Similarly, oligodendrocytes have higher iron content and lower levels of glutathione and its related antioxidant enzymes, making them vulnerable to oxidative injury. After SCI, a cascade of oxidative events is initiated by reactive oxygen species causing a combination of necrosis and apoptosis, resulting in degeneration of gray matter, and interfering with local spinal circuits within the injury center. In the following few days to weeks, inflammatory cells and phagocytes remove injured neurons, their detached axons and extracellular elements of the necrotic core at the site of injury leading to the formation of fluid filled cysts. These cysts also known as areas of myelomalacia are found in 30% of patients resulting in posttraumatic syringomyelia. In the mean time, axonal degeneration begins along the distal section of the transected axon.

### Role of glutamate and excitotoxicity

Exposure of neurons and oligodendrocytes to increased concentrations of the amino acid glutamate, an excitatory neurotransmitter, leads to cell death referred to as excitotoxicity. It has been hypothesized that increased activation of glutamate receptors may be the factor leading to the bereavement of neurons. Under physiological conditions, glutamate is readily taken up by cells decreasing the extracellular concentration. Models involving exogenous administration of glutamate have shown limited injury, indicating that it is readily taken up decreasing the concentration to tolerable levels. However, cytotoxic levels of glutamate have been noted after SCI. Studies have shown that these uptake systems may reverse during injury leading to a further release of glutamate in the post injury period. The effect of exogenous glutamate on uninjured spinal cord has also been shown to induce neuronal death. A study conducted at the University of Texas involving the administration of different concentrations of glutamate in the spinal cords of Sprague–Dawley rats showed evidence that glutamate at the levels observed post SCI contributes to the secondary injury; further dose dependant damage was observed suggesting a receptor-mediated process. The study observed lasting functional impairment as observed in an activity box and on the Basso-Beattie-Bresnahan (BBB) scale (discussed later).[Table 1]

**Table 1:** Summary of the Basso, Beattie and Bresnahan locomotor rating scale for open field testing in rats.

0	No observable hind limb (HL) movement.
1	Slight movement of one or two joints, usually the hip and/or knee.
2	Extensive movement of one joint or extensive movement of one joint and slight movement of one other joint.
3	Extensive movement of two joints.
4	Slight movement of all three joints of the HL.
5	Slight movement of two joints and extensive movement of the third.
6	Extensive movement of two joints and slight movement of the third.
7	Extensive movement of all three joints of the HL.
8	Sweeping with no weight support or plantar placement of the paw with no weight support.
9	Plantar placement of the paw with weight support in stance only (i.e., when stationary) or occasional, frequent, or consistent weight supported dorsal stepping and no plantar stepping.
10	Occasional weight supported plantar steps, no forelimb (FL)-HL coordination.
11	Frequent to consistent weight supported plantar steps and no FL-HL coordination.
12	Frequent to consistent weight supported plantar steps and occasional FL-HL coordination.
13	Frequent to consistent weight supported plantar steps and frequent FL-HL coordination.
14	Consistent weight supported plantar steps, consistent FL-HL coordination; and predominant paw position during locomotion is rotated (internally or externally) when it makes initial contact with the surface as well as just before it is lifted off at the end of stance or frequent plantar stepping, consistent FL-HL coordination, and occasional dorsal stepping.
15	Consistent plantar stepping and consistent FL-HL coordination; and no toe clearance or occasional toe clearance during forward limb advancement; predominant paw position is parallel to the body at initial contact.
16	Consistent plantar stepping and consistent FL-HL coordination during gait; and toe clearance occurs frequently during forward limb advancement; predominant paw position is parallel at initial contact and rotated at lift off.
17	Consistent plantar stepping and consistent FL-HL coordination during gait; and toe clearance occurs frequently during forward limb advancement; predominant paw position is parallel at initial contact and lift off.
18	Consistent plantar stepping and consistent FL-HL coordination during gait; and toe clearance occurs consistently during forward limb advancement; predominant paw position is parallel at initial contact and rotated at lift off.
19	Consistent plantar stepping and consistent FL-HL coordination during gait; and toe clearance occurs consistently during forward limb advancement; predominant paw position is parallel at initial contact and lift off; and tail is down part or all of the time.
20	Consistent plantar stepping and consistent coordinated gait; consistent toe clearance; predominant paw position is parallel at initial contact and lift off; tail consistently up; and trunk instability.
21	Consistent plantar stepping and coordinated gait, consistent toe clearance, predominant paw position is parallel throughout stance, consistent trunk stability, tail consistently up.

Originally published in Journal of Neurotrauma, Volume 12, Number 1, 1995.

## LACK OF RECOVERY AND REGENERATION

Several factors play key roles in inhibiting regeneration of axons. These include glial scar formation and several inhibitory proteins such as CSPGs, Nogo-A, myelin-associated glycoprotein (MAG) and oligodendrocyte myelin glycoprotein (OMgp).

### Chondroitin-Sulfate Proteoglycans (CSPG)

CSPGs are inhibitory extracellular matrix molecules that are upregulated after injury to CNS. CSPGs are composed of large molecular complexes and consist of a central protein core to which highly sulfated glycosamnoglycans are attached. Enzymatic separation of the glycosaminoglycans from protein core significantly reduces the inhibitory effect of glial scar. In animal trials carried out at the University of Cambridge and King’s College London, agents such chondroitin sulfate proteoglycan-degrading enzyme chondroitinase ABC have shown to promote axonal sprouting after SCI in rats.

### Myelin/Oligodendrocyte Inhibitory Proteins

Myelin/oligodendrocyte associated inhibitory molecules have been studied extensively. Nogo-A, B, and C constitute the Nogo family which are glycosylated oligodendrocyte transmembrane proteins. Nogo-A consists of two structural domains, a 66-base amino acid loop termed as Nogo-66 common to all three Nogo isoforms and a large stretch termed as NiG or central inhibitory domain of Nogo-A which is specific for Nogo-A only. Growing axons when exposed to Nogo-A are known to retract and collapse axonal growth cones. This inhibitory activity of Nogo-A is due to the presence of the NiG domain.

The Nogo receptor (NgR) is known to bind to the 66-base amino acid loop (Nogo-66). Other axonal growth inhibitory proteins such as MAG and OMgp also bind to the NgR and use a common signalling mechanism to inhibit axonal growth. Efforts to overcome the inhibitory effect of Nogo by targeting the Nogo-66 receptor are in progress. A study on rats carried out at Yale University School of Medicine (USA) has shown that the application of Nogo-66 receptor antagonist peptide NEP1–40 (Nogo extracellular peptide, residues 1– 40) promotes recovery after SCI by extensive growth of corticospinal axons, sprouting of serotonergic fibers, upregulation of axonal growth protein SPRR1A (small proline-rich repeat protein 1A), and synapse re-formation. Another study showed that binding of the MAG to NgR was inhibited by casein kinase II (CK2) mediated phosphorylation of NgR. This inhibition of MAG binding to NgR suppressed the signalling by NgR and allowed neurite regeneration to take place. All these studies highlight the role of myelin/oligodendrocyte inhibitory molecules in inhibition of regeneration in axons and the importance of targeting the NgR when dealing with SCI.

### Scar formation

Despite the existence of endogenous neural stem cells in the adult spinal cord, neurogenesis does not occur, in normal or injured states during adulthood. However, it has been noted that after the spinal cord is damaged, undifferentiated nestin positive cells arise from around the central canal in the vicinity of the lesion. These cells are known to proliferate dynamically, and migrate to the site of the lesion where they differentiate into astroglia. Approximately all of the cells differentiate into astroglia forming scar tissue with the passage of time. LIF and CNTF stimulate the differentiation of neural stem/progenitor cells into astroglia by activating the gp130, JAK, signal transducers and STAT pathway. These reactive astroglia also express CSPG which inhibits axonal growth and inhibit axonal regeneration. Formation of the astroglial scar is a major hindrance to regeneration of disrupted neuronal axons, extension of axons past the injury site and their remyelination.

Evidence that the glial scar plays a major role in antagonizing neuronal sprouting after SCI comes from the identification of CSPG as an axonal growth-inhibiting molecule expressed by reactive astrocytes. Furthermore, this idea is strongly supported by studies carried on mutant mice lacking the genes encoding glial fibrillary acidic protein (GFAP) (an intermediate filament protein) and vimentin, highly expressed by reactive astrocytes. The mutant mice showed impaired scar formation accompanied by improved axonal development and better functional recovery. This may be attributable to suppression of the astrocytic response.

Formation of glial scar is an endogenous attempt of the CNS by glial cells to restrain the site of injury and promote healing. Apart from the reactive astrocytes, the oligodndrocyte precursor cells, microglia and macrophages also contribute to the formation of glial scar. As with astrocytes, oligodendrocyte precursors are also associated with upregulation of CSPGs, a major contributor to the inhibitory properties of the adult CNS glial scar.

### Beneficial role of the glial scar during acute phase

**Figure 1:**
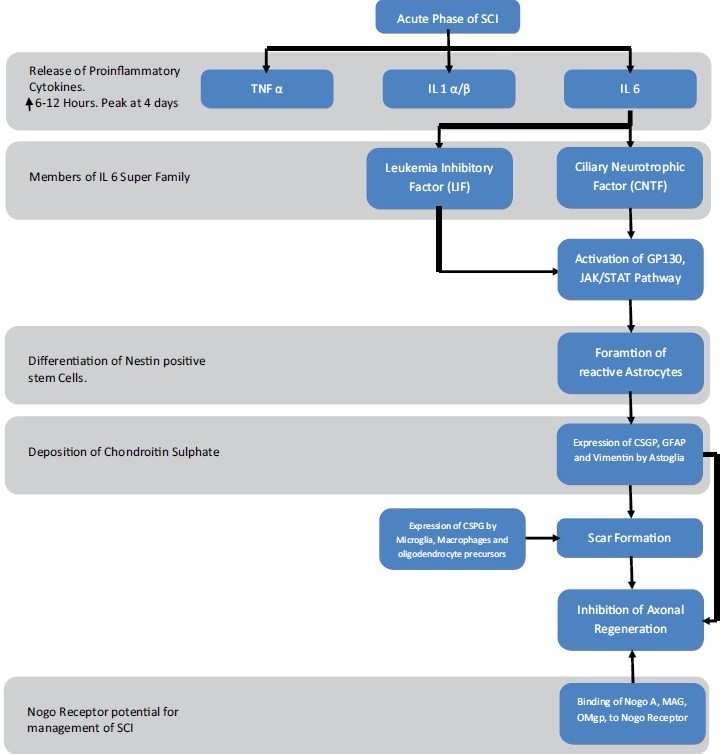
Summary of events in the acute phase of SCI

Although it is widely hypothesized that the glial scar is a hindrance to the treatment of SCI, some studies have shown a useful role of the glial scar during the acute phase of SCI.[Figure 1] The removal of reactive astrocytes or prevention of their migration and scar formation after injury led to cessation of repair along the blood-brain barrier causing massive infiltration of inflammatory cells thereby augmenting the loss of neurons and oligodendrocytes and a poorer functional recovery. [Table 2] Furthermore, trials involving transgenic mice with improved astrocyte migration and premature scar formation showed facilitated recovery. The importance of an acute astrocytic response to limit and control the inflammatory response should not be under estimated and it should be kept in mind that the formation of glial scar is of vital importance during the acute phase even at the cost of reduced axonal sprouting subsequently. It is for this reason that most studies recommend transplanting cells one-two weeks after injury.

**Table 2:** Summary of hypothesized mechanisms of discussed sources of stem cells and their possible role in the repair of injured spinal cord

Source	Hypothesized mechanisms	Functional effects	Possible role in SCI Repair
Cord blood derived stem cells			
	↓ Apoptotic genes		↓ Apoptosis.
	↓ FaS	Blocks activation of caspases 3 and 8.	↓ Apoptosis.
	↓ tPA	Stops degradation of MBP. Stops initiation of MMP cascade.	↓ Demylination. ↓ Scar formation.
Placental stem cells			
	↑ IL-10	↓ Lymphocyte proliferation.	↓ 1mmune response.
	↑ VEGF	Angiogenesis.	
Olfactory ensheathing cells			
	↑ FGF	Formation of fibroblast sheath.	Axonal regeneration by protecting from inhibitory effects of microenvironment.
	↑ NGF		Axonal regeneration and sprouting.
	↑ BDNF		
	↑ GDNF		
	↑ CNTF		
	↑ VEGF	↑ Angiogenesis.	
Bone marrow stromal cells			
	↑ NGF		↑ Axonal regeneration and sprouting.
	↑ BDNF		
	Nestin positive ependymal neural stem cells	Neural stem cells months after injury	Regeneration of chronically injured spinal cord

### Current Approaches to Management of SCI

SCI may result in severe sensory and motor deficits at and below the level of injury ranging from incomplete (myelopathy/paraparesis/quadriparesis) to complete (paraplegia/quadriplegia) deficits. The actual damage is exacerbated by the immune response, creating a microenvironment that has been shown to cause apoptosis and damage to surrounding functional neurons. Therefore, any potential method for functional restoration must address the inhibitory microenvironment along with the re-establishment of ascending/descending tracts and local synapses. Recently, efforts have been made to contain the local response; this includes delivery of neurotrophic factors, interleukins, and agents that promote survival and neuronal growth and enable the neural tissues to establish connections. Some studies have even proposed filling the gaps of myelomalacia with biodegradable/ non biodegradable scaffolds in order to guide axons. Efforts have also been made to establish new synapses and provide a conducive environment, by grafting neural tissue from autologous and fetal sources including embryonic or adult stem cells, Schwann cells, genetically modified fibroblasts, bone marrow stromal cells (BMSCs), and olfactory ensheathing cells (OECs) and combinations/ variants thereof.

Stem cells have been under constant research due to their potential clinical applications in multiple diseases. Their ability to differentiate into various cell lines replacing damaged or non functional tissue can potentially improve the outcome of many diseases; however, the ethical and moral issues surrounding the source of these cells and the limitations in their extraction, culture and transplant are major setbacks. Neurally differentiated stem cells have already been explored as treatment options in various degenerative CNS diseases including multiple sclerosis, amyotropic lateral sclerosis and Parkinson’s disease. Trials of stem cell therapy in the PNS are also under way, a recent trial claimed improved paw function in rats with brachial plexus injuries after OEC transplant. Here we review the various sources of neural stem cells including cord blood, placenta-derived stem cells, OECs, embryonic stem cells, and bone marrow stromal cells and their potential application in the management of SCI.

### Cord Blood

Umbilical cord blood stem cells (UCBSCs) initially considered only under hematological uses for replacement of hematopoietic cells have now been shown to differentiate into a large number of lineages. It is easily accessible, devoid of ethical issues and potentially the largest source of human stem cells. They are currently employed in the treatment of hematopoietic disorders where bone marrow donors are unavailable. It is estimated that 1 million pluripotent cells are present in an average cord blood volume of 80 ml; however, most of these cells are directed to a lineage of hematopoietic cells and a high cell concentration is required for initial plating.

Recently human UCBSCs have been shown to differentiate into neural cells *in vivo* and *in vitro*. Culture of pluripotent cord blood cells with retinoic acid(RA) and brain-derived neurotrophic factor (BDNF) is recommended for differentiation along neural lineages. Dibutyryl cyclic AMP has shown to bring about terminal differentiation of UCBSCs into neuronal cells. Researchers have successfully differentiated UCBSCs into neuronal cells on animal serum media with different combinations. They include Dulbecco’s modified Eagle’s medium (DMEM) augmented with 10% fetal bovine serum (FBS), FBS with fetal calf serum and endothelial growth factor (EGF), 10% FBS, granulocyte-macrophage colony-stimulating factor (GM-CSF) or L-glutamine. Controlled media containing DMEM, B27, N2, BDNF, EGF, nerve growth factor (NGF), RA, dBcAMP, collagen and fibronectin have also showed success.

Cryopreseved UCBSCs have been demonstrated to retain their capacity to differentiate along neuronal cell lines. Thus UCBSCs can be cryopreserved at birth for possible use in future to provide the most compatible HLA type.

Animal trials have shown promising results. Significant increases in locomotor responses were noted in Sprague–Dawley rats after transplantation of purified human UCBSCs and BDNF in rats with moderate SCI at T8-T10. BBB locomotor rating scores were used to access the outcome, the standardized 21 point scale is based mainly on hind limb movement and locomotor efficiency. The BBB score for a normal rat considered is 21 with lower scores indicating functional impairment. The results indicated that the BBB scores increased significantly from 6.2 immediately after injury. At eight weeks post transplantation, the final BBB scores were 11.8 in the group receiving purified UCBSC injection and 13.5 in the group receiving purified UCBSC and BDNF therapy, compared to 11 in the control group. A similar study transplanted neurally differentiated UCBSCs cultured in RA and human EGF into male Lewis rats with moderate SCI at T10, the transplanted group showed significantly improved BBB scores of 15.78 compared to 6.54 for the control group. This study showed much better functional recovery compared to the previous study in which UCBSCs were not differentiated into neuronal lines prior to transplantation. Another study, carried out on 62 Sprague–Dawley rats with weight drop SCIs at T9, also showed functional improvements on transplantation of neurally induced UCBSCs at the epicenter of injury. Although the mechanism of UCBSC-mediated improvement is unknown, it is hypothesized that the functional improvement is attributable to the ability of UCBSCs to down regulate apoptotic genes, fas, and tissue plasminogen activator discussed earlier.

A case study from the Seoul National University (Korea) has reported transplantation of cord blood cells in a 37-year-old female patient with SCI at T12 due to a fall causing a fracture at T11 and T12 vertebrae. The patient was paraplegic after emergency surgery but transplantation of UCBSCs after 19 years and 6 months improved sensory and motor function. She was able to maintain an upright posture on post operative day (POD) 13 and was able to raise her legs by about 1 cm on POD 15. Her hip flexor function gradually improved till POD 41 and dermatomal somatosensory-evoked potential was recovered to L2 in both legs by POD 41. Approximately 3 million multipotent stem cells were transplanted into this patient. Such a dramatic improvement almost 20 years after injury, coupled with a lack of subsequent cases reproducing similar results can be considered anecdotal at best.

### Placenta Derived Stem Cells

Stem cells derived from the fetal membranes of the full-term placenta have been considered as a source with an abundant supply and fewer ethical and moral issues. Placenta-derived stem cells (PDSCs) have been shown to differentiate into osteoblasts, adipocytes, chondrocytes, myocytes, endothelial cells and neurons. A recent study showed that human PDSCs cultured with rat brain cells, RA or 1-methyl-3-isobutylxanthine (IBMX) differentiated into neuronal cells. Using a culture medium including ascorbic acid (AA), along with RA, and IBMX PDSCs have been differentiated into dopaminergic neurons which expressed dopaminergic markers and secreted the neurotransmitter.

PDSCs have been shown to suppress immune response by inhibiting lymphocyte proliferation and increasing angiogenesis. These are thought to be mediated by the release of Interleukin 10 and vascular endothelial growth factor (VEGF). The immunomodulatory potential of fetal placental stem cells was shown to be higher than that of maternal placental stem cells. It has been hypothesized that the immunomodulatory and angiogenetic effects of placental stem cells lead to functional improvement in SCI; however, no animal or human trials have been published yet.

### Olfactory Ensheathing Cells

The olfactory bulb houses numerous primitive stem cells (olfactory ensheathing cells; OECs) which continuously regenerate odor-detecting nerves. Because these cells are found in a fairly accessible region of the brain and can be removed from a person’s olfactory bulb without causing any significant harm to the patient, they have been considered as a prospective non-embryonic source for cells avoiding the stigma associated with other stem cell lines. Another added advantage is that they can be extracted as autografts from the patient, decreasing the immune response against transplanted cells or as allografts.

Lately OECs have attracted much interest because of their potential for transplantation-based therapy in CNS diseases. Extensive studies on transplantation of OECs from the nasal mucosa have shown promising results. The precise cellular mechanisms underlying functional improvement after OEC transplantation are not fully understood; however OEC has been shown to secrete varying amounts of NGF, BDNF, GDNF, CNTF, FGF and VEGF, depending on culture conditions or *in vivo* microenvironment. Hence the improvement noticed maybe attributed to their ability to remyelinate and regenerate damaged axons and promote angiogenesis by secreting growth factors. It seems that the role of OECs in spinal cord repair is more supportive than replacing actually replacement of lost neurons.

OEC transplantation into transected spinal cords of Sprague–Dawley rats shows bridging of the lesions in a unique pattern. A sheath of fibroblasts surrounds a group of regenerating axons; this sheath is believed to protect the regenerating axons from the inhibitory microenvironment of the lesion.

Keeping in mind that OECs mainly provide a supportive role in axonal regeneration, trials with combined transplantations of neural stem cells and OECs are already underway. A trial carried out on 40 female Wistar rats at the Tehran University of Medical Sciences (Iran) transplanted embryonic stem cell-derived motor neuron (ESMN) along with OECs. ESMNs were cultured by exposing mouse ES cells to RA while the OECs were obtained from olfactory nerve rootlets and olfactory bulbs. The cotransplantation had a synergistic effect, promoting neural regeneration along with ESMN survival and partial functional recovery. The cotransplant group showed better BBB scores of 8.5 four weeks post transplant when compared to 7.33 with OECs only, 7.5 with ESMNs only and 0.66 in the control group. However, the difference observed between the transplant groups was not statistically significant.

Another promising study carried out at the Sun Yat-sen University (China) tested the efficacy of co-grafting human BMSC and OEC in treating SCI in rats. The co-graft led to better functional recovery and higher gait scaling in comparison to the groups receiving mono-therapy. Larger axon bundles were also noted through the transitional zone between the normal and injured regions in the group receiving co-therapy. Thus, the combined use of BMSC and OEC may provide an improved approach for the treatment of SCI.

Apart from co-grafting with other stem cell lines, trials undertaking co-therapy with OECs and various non cellular therapies have also been carried out. The department of orthopaedics at the Second Hospital of Xi’an Jiaotong University (China) conducted a study combining OEC transplants with chondroitinase ABC therapy. The combination was effective in the repair of SCI in Sprague–Dawley rats to some extent and presents a new direction in which further approaches can be made. The co-therapy group showed significantly improved functional outcome measured by BBB scores when compared to the control and mono-therapy groups. The maximal transverse diameter and area of necrosis was also significantly reduced along with the expression of GFAP in the co-therapy group.

Another study conducted at the University of British Columbia (Canada) on Sprague–Dawley rats undertaking co-therapy with BDNF and OEC transplantation showed decreased axonal regeneration and functional recovery in a food-pellet reaching test and a cylinder test when compared to mono therapy with either. The mechanism for this decreased response was not understood, but enhanced sprouting of calcitonin gene-related peptide-positive axons was observed rostral to the lesion. *In-vitro* testing of hypothetical synergism between different cell lines and therapies is highly recommended before any interventional study in human subjects.

Human trials have been carried out in some countries. Recently, a Phase I/IIa trial was conducted in by the National Centre for Adult Stem Cell Research, Griffith University (Australia). Their main aim was to test the practicability and safety of transplantation of autologous OECs into the injured spinal cord in human. Autologous, cultured OECs were transplanted into the spinal cords of six patients with complete, thoracic paraplegia for at least two years. The results showed that the transplant was safe and there were no adverse findings three years after the transplant; however, no functional or sensory improvement was noted in the participants.

A more extensive clinical trial was carried out at the Beijing Hongtianji Neuroscience Academy (China) to analyze the therapeutic effect of olfactory ensheathing cells (OECs) transplantation on CNS diseases. Out of the 1,255 participants from 71 countries or regions, 656 participants suffered from SCI, and the rest with other diseases including amyotrophic lateral sclerosis (457), cerebral palsy (68), multiple sclerosis (20), the sequelae of stroke (11), ataxia (10), and residual diseases (33). The patients were transplanted with fetal olfactory bulbs which were donated voluntarily after accidental abortions. The cells were cultured for two weeks before transplantation. One hundred and twenty patients with SCI received over one year follow-up showed some improvement in their ASIA scores for sensory-motor functions highlighting the fact that OECs transplantation into brain and spinal cord maybe feasible and safe.

### Embryonic Stem Cells

Human embryonic stem cells (hESCs) are considered to be an abundant source for pluripotent human stem cells; however, ethical, moral and religious limitations present hindrances to the use of embryonic stem cells in research and medicine. These cells demonstrate higher levels of telomerase, an enzyme preventing cell senescence, hence multiple generations of cells can be cultured. Recent studies, however, suggest that replication over 80 generations caused cell lines to accumulate mutations. hESCs have the potential to generate along any cell line depending on their isolation, level of differentiation, handling and culture environment.

The culture environment requires paracrine and autocrine factors along with physical contact via cell-surface adhesion molecules and gap junction mediated intercellular coupling. Morphologically similar hESCs differ physiologically; thereby displaying variable differentiation potential. Recent studies suggest that HUES-9, a physiological subtype of hESCs, preferentially differentiates along neural lineages (i.e. neurons, oligodendrocytes, astrocytes and gangliosides).

Neurally differentiated embryonic stem cells possess limitations due to rapid cell death in serum free conditions. Culture of ESCs in relative hypoxia (4% O_2_) after initial culture at 20% O_2_ has shown to decrease cellular apoptosis through inhibition of apoptosis initiating factor. This down regulation of apoptosis in low O_2_ tension can be related to the *in vivo* environment in which neural stem cells form the embryonic neural system. Low O_2_ culture maintains a certain proportion of stem cells as undifferentiated cells but it has no effect in preventing stem cells from differentiating along neural lineages.

An animal trial on rats with midline lateral hemisection models of SCI at The Cell Science Research Center, Royan Institute (Iran) showed improved recovery of hind limb locomotor function after transplantation of hESCs in collagen scaffolds. Another trial carried out at the National Chung Hsing University (Taiwan) on Sprague–Dawley rats with spinal lesions at T8-T9 showed that administration of GM-CSF along with hESC transplant had a better functional outcome, motor evoked potentials, and conduction latency when compared to those receiving mono-therapy with either. Higher expression of neuronal maturation markers neuronal nuclear antigen (Neu-N) and microtubule-associated protein 2 (MAP-2) was also observed in those receiving a combination of hESC and GM-CSF. In view of these promising results, the USFDA has granted permission to Geron Corporation for conducting phase I trials for embryonic stem cells in various diseases including SCI.

Despite promising findings, a major factor in transplant of neural stem cells is termination of growth; initially the neuronal growth causes functional improvement but it soon leads to tumor formation. A recent animal study at the Nara Medical University (Japan) indicates that co-transplantation of neural stem cells with bone marrow stromal cells (BMSCs) prevents tumor formation without compromising functional recovery. In these experiments on mice with SCI, transplanted hESCs developed tumors at the graft site and functional improvement ceased in three weeks; in contrast no tumors or cessation of improvement was observed in mice receiving BMSCs along with hESCs. *In vitro* results showed that BMSCs secreted NGF, GDNF, and BDNF for five weeks, inducing the differentiation of undifferentiated hESCs along neural lineages and preventing tumor formation.

### Bone Marrow Stromal Cells

Adult bone marrow is a source of stem cells (Bone Marrow Stem Cells; BMSCs) that have been shown to differentiate along a variety of cellular lines, including osteocytes, chondrocytes, myocytes, hepatocytes, epithelial linings, glia, neurons and Schwan cells. A recent study indicates that β-mercaptoethanol (BME) followed by NGF induces BMSCs to express neural markers and differentiate along neural lines. They have also been shown to express an array of growth factors and cytokines to support sprouting axons. BMSCs possess many advantages over other sources as immunologic rejection is minimal and auto transplants leave very few ethical concerns. It is due to these advantages that BMSCs are the main center of attention in stem cell based therapy in many neurological diseases including SCI. In an average bone marrow harvest, only 0.125% of the cells are BMSCs, and an age-dependant inverse correlation with number of cells isolated in the first passage has also been demonstrated. However, it has also been noted that sufficient BMSCs can be successfully cultured for an auto transplant from SCI patients.

Keeping in mind the limited availability and need for optimum delivery into the CNS, the effectiveness of various BMSC delivery routes including intravenous (IV), intra-arterial (IA) intrathecal and lumbar puncture (LP) has been explored in stroke, traumatic brain injury and SCI models. A trial carried out at Henry Ford Hospital (Michigan, USA) involving the IV administration of BMSCs to Wistar rats 24 h after a traumatic brain injury (TBI) showed that the BMSCs preferentially migrated to the site of injury and increased the expression of growth factors including NGF and BDNF leading to significantly decreased sensory and motor deficits. Deposits of BMSCs were also found along the other organs but localized to their vascular structures, without any obvious adverse effects. Another study carried out by the same team shows BMSCs injected into the internal carotid artery of male Wistar rats with impact induced TBI showed that BMSCs migrate to the site of injury site, suggesting that intra-arterial transplantation of BMSCs could be a practicable route of administration in treating injured brain tissues. A study from Drexel University College of Medicine (Philadelphia USA) claims LP deliveries show significantly better cell engraftment and tissue sparing with reduced immune response when compared with IV delivery in SCI. However another trial from Lorestan University of Medical Sciences (Iran) claims similar functional outcomes in IV and intraspinal deliveries. A magnetic targeting system has shown to concentrate BMSCs labelled with magnetic beads by placing magnets in the paravertebral muscles at the target level.

The ease of availability and culture of BMSCs in comparison to other cell sources has led to a lot of interest in using BMSCs for treating SCI and many animal trials have been carried out. A trial from the Neuroscience Research Unit of the Mapfre-Medicine Foundation (Spain) involving chronic paraplegic (three months) female Wistar rats with impact drop injuries at T6-T8 vertebral levels not only demonstrated tissue bundles bridging the centromedullary cavity but also showed significant functional improvement. Thirty days post transplant, the BBB scores of the transplant group had improved to 8 from an initial of 0, whereas no improvement was noted in the control group. At the end of the 30 day period, the improvement had not plateaued but the animals were sacrificed for histological examination.

A recent trial from Southern Medical University (China) involving co-administration of purified bone marrow stromal cells and granulocyte colony stimulating factor (G-CSF), known to mobilize hematopoietic stem cells and inhibit neural cell apoptosis, in female Sprague–Dawley rats with micro-scissor incisions at T9-T10 showed significant improvement in BBB when compared to the control group. On histological examination, a decreased number of apoptotic cells and increased expression of neural-cell markers including neuron-specific enolase, GFAP, and neurofilament was observed around the site of injury in contrast to the groups that received only BMSCs or G-CSF. Keeping these results in mind, a better therapeutic effect can be expected with BMSCs and G-CSF co administration.

Trials in larger animals like pigs have also been done after SCI induced by the application of two Heifetz’s clips at T12-T13 for 30 min. Out of the 10 pigs used in this trial, 7 received autologous BMSCs in autologous plasma whereas 3 received only autologous plasma and were considered as controls. They showed signs of functional recovery three weeks post transplant by grunting on stimulation of posterior extremities, which progressed to signs of movement at four weeks. Electrophysiological studies three months post-SCI showed somatosensory-evoked potentials in the transplant group, which had been absent initially. MRI studies at three months revealed a decrease in the centromedullary cavity volumes and histological analysis also revealed axonal sprouting.

The appropriate time of stem cell transplant after SCI regardless of the source has always been a continuous topic of debate warranting what should be considered the best time for a transplant. A trial on 121 female Sprague Dawley rats, showed significantly improved tissue sparing with early transplant of BMSCs indicating their neuroprotective role in early spinal cord injury. Therefore, it is recommended that any trial being developed should consider the role of early transplants during the acute phase of the injury.

Recently, the case of a 37-year-old male quadriplegic patient who sustained a SCI at C4-C5 vertebral level as he fell from a height of 7m has been reported from Kansai Medical University (Japan). BMSCs were collected from his ileum, on the third day after injury and cultured for 10 days. BMSCs suspended in saline were infused through a lumbar puncture on day 13. His ASIA scores for motor and sensory levels showed some gradual improvement at one and three months after transplantation, but no further sensory improvement was noticed at six months. However ASIA scores evaluated at such an early stage would not be reliable due to a gradual improvement in the patient due to settlement of spinal shock and associated symptoms. The patient is reported to be gradually improving and able to sit on a wheel chair and drive it slowly by himself. The investigators recommend that transplantation of BMSCs should be carried out as soon as possible after injury, and for optimum results should be carried out within three weeks of injury; keeping in mind that the BMSCs will take 7-10 days to multiply and reach an adequate density for transplantation.

BMSCs are proving to be the most attractive option for researchers among the sources of stem cells and are attracting a lot of attention as future options for treatment of various neurological diseases. BMSCs show great promise for the treatment of SCI warranting further investigation in this direction.

## CONCLUSION

Stem cell transplantation as a strategy for spinal cord regeneration is at the forefront now. The animal studies and *in vitro* studies provide a solid platform to proceed to well-designed human studies on stem cell transplantation for spinal cord injury. More than two decades ago, optimism prevailed with the discovery of molecules in mature CNS that inhibited neurite growth. Unfortunately, the problem is multi-factorial and the answer too lies in a multi-factorial approach. It will require choosing the right source of stem cells, priming them appropriately and transplanting them with right conditions and factors to cajole them into doing the job. Of all the stem cells reviewed in this article, olfactory ensheathing cells seem to be the most promising; bone marrow stromal cells are strong candidates for an adjunctive role. The key strategy in developing the therapeutic basis of stem cell transplantation for spinal cord regeneration is to weed out the pseudo-science and opportunism. All the trials should be based on stringent scientific criteria and effort to bypass that should be strongly discouraged at the international level.
